# One Health Economics 2.0 – commentary

**DOI:** 10.1080/11287462.2025.2511517

**Published:** 2025-05-28

**Authors:** Zohar Lederman

**Affiliations:** Department of Emergency Medicine, University of Hong Kong, Pok Fu Lam, Hong Kong

Ben Capps makes a case for a One Health (OH) economy in research, defined as a lens through which humans should devise and evaluate research initiatives. In contrast to the status quo, a OH economy means that research, not only biomedical research, should be devised and conducted with the potential risks and benefits towards all involved parties in mind. This includes humans, non-human animals, and the environment. Capps uses space exploration as an example: currently space exploration, particularly by private entities but also by governmental ones, is being conducted for the sake of human interests, both present and future. But this lens is too narrow, argues Capps. Instead, viewing space exploration through a OH economy lens means that we should allocate funding for it based on its potential risks and benefits towards not only humans but also animals present and future as well as ecosystems present and future.

This work continues Capps’ advocacy for, and examination of OH environmentalism, or the idea that environments matter because they enable and sustain the rights of right bearers. These right bearers are not limited to humans. It is here, then, that environmental philosophy, economics, bioethics, and research ethics intersect: figuring out who has rights and thus noteworthy interests in a particular context, and deducing the corresponding duties of others. “Others” here denote only those who can at least potentially understand the concept of morality and duties towards rights holders, meaning moral agents. As far as science allows us to know currently, this title is restricted to some human beings. OH environmentalism, in fact, while being about all right bearers, also known as moral patients, is not *for* them; it is meant only *for* moral agents. In other words, OH ought to benefit and respect all creatures with moral value who deserve respect – in bioethics, these are commonly signified as persons, consisting of all human beings and some other, non-human animals. The demand to follow the dictates of OH, however, only oblige those creatures who can morally reason, judge, and act based upon this moral judgement, i.e. some human beings.

The bulk of the scientific literature on OH seems to be nothing other than traditional public health and biomedical research, simply calling for sectoral de-siloing and interdisciplinary collaboration to improve public health, which by definition is limited to the health of humans (Bègue et al., 2025). Even literature that proclaims to discuss the ethics of OH has been either overly superficial (Goldberg and Patz, 2015) or has focused on community engagement, which again readily applies to public health research but not uniquely to OH research per se (Nguta et al., 2022). Capps’ proposal is novel in using a OH framework to expand the ethical scope of research to include environmental considerations. It is also novel in referring to all kinds of research rather than focusing on biomedical research.

Acknowledging the moral worth of animals may but need not result in a total ban on animal use in biomedical research (Regan, 2004). Rather, it means that researchers and other stakeholders ought to be much more careful and systematic about vetting biomedical research from an ethical perspective that includes but is not limited to scientific merit (Beauchamp and DeGrazia, 2020). Similarly, adopting OH as a genuine attempt to accommodate animals and ecosystems entails much more serious and explicit efforts to ethically balance tradeoffs among these objects of moral worth (Lederman, 2024; Adisasmito et al., 2022). During the Ebola Virus outbreak in West Africa in 2013–2016, for instance, authors have made an ethical argument to vaccine apes to benefit both humans and apes, thus achieving shared benefits (Capps and Lederman, 2015a; Capps and Lederman, 2016). There is currently some move in the scientific OH literature in that direction, but it is not as explicit as it should be, and it is often expressed merely as a scientific necessity rather than an ethical one (Lam et al., 2024).

Indeed, despite the recent introduction of a prominent OH ethicist into the OH High-Level Expert panel (One Health High-Level Expert Panel (OHHLEP), n.d.), the chasm between the science of OH and the ethics of OH seems to be expanding. The notion and alleged spirit of OH will increasingly appear in scientific publications and governmental policies, but bioethicists who can actually think critically about the ethics involved in engaging OH research according to the proclaimed goals and spirit of OH will be excluded. One example for this is the ever-growing ratio of OH journals and publications to the inclusion of ethicists in these publications, professional OH meetings, and generally to collaborative research with ethicists (Lederman, n.d.). This would eventually result in OH actual research and practice being made redundant with traditional public health and/or other titles such as EcoHealth, Planetary Health etc. In other words, even though OH was originally framed as a biomedical approach that aims to benefit animals and the environment as well as humans, it would end up being geared towards the health of humans, thus not really offering anything conceptually novel beyond traditional public health and Ecohealth or Planetary Health that explicitly just aim to benefit human communities.

Or we can start being serious about OH ethics. Capps appeals to the idea – coined initially by him – of Universal Goods (Capps and Lederman, 2015b). Markets provide goods, which satisfy human preferences. But paraphrasing an old attaché, markets cannot buy rights and are indeed restricted by them. The same way markets and the research driven by markets are limited by inviolable human rights, markets and research endeavors should be limited by and made subservient to the rights of all right-bearers.

This OH economy reframes a right to science and inquiry, which means that the right-bearer is entitled to the benefits of scientific inquiry and more generally to access the “knowledge commons,” the pool of knowledge that is created by scientific inquiry and is shared by a given community of rights (Gewirth, 1996). In this way, OH links to moral rights and epistemic justice. A right to science, or a right to “know,” in turn can drive markets. Markets, then, are both driven and drive research, and rights (should) drive and restrict both.

Conceptualizing a right to science in this OH fashion has wide-ranging implications. While Capps focuses on science exploration, I will bring the discussion back to biomedical research. Consider animal laboratory research. One of the prevailing arguments to justify such research is the lack of alternatives. This lack of alternatives, however, is usually accepted as truism and rarely explored or challenged further. This lack of alternatives however – if alternatives indeed do not exist – results from a specific anthropocentric conceptualization of the right to science that in turn drives markets. Such moral anthropocentrism essentially means that animals are, at the end of the day, expendable for human benefit. The same way markets and research exist because some right-bearers want them to exist, research into, and markets of alternatives to animal research will come to exist if some right bearers will want them to exist. A OH approach that takes OH ethics seriously would include all right bearers in driving the search for alternatives, and will plausibly accept animal lab research only if conducted within a therapeutic framework, meaning only if it benefits the animals being experimented on or at least if it can potentially benefit other non-human animals.

With this publication then, as with others (Capps, 2022a; Capps, 2022b; Capps, 2024), Capps leads the way in OH ethics and challenges both ethicists and scientists who proclaim to engage OH to think more critically about their practice and the real implications, both practical and philosophical, of OH.
